# The use of induced pluripotent stem cells to reveal pathogenic gene mutations and explore treatments for retinitis pigmentosa

**DOI:** 10.1186/1756-6606-7-45

**Published:** 2014-06-16

**Authors:** Tetsu Yoshida, Yoko Ozawa, Keiichiro Suzuki, Kenya Yuki, Manabu Ohyama, Wado Akamatsu, Yumi Matsuzaki, Shigeto Shimmura, Kohnosuke Mitani, Kazuo Tsubota, Hideyuki Okano

**Affiliations:** 1 Laboratory of Retinal Cell Biology, Department of Ophthalmology, Keio University School of Medicine, 35 Shinanomachi, Shinjuku-ku 160-8582, Tokyo, Japan; 2 Department of Ophthalmology, Keio University School of Medicine, 35 Shinanomachi, Shinjuku-ku, Tokyo 160-8582, Japan; 3 Division of Gene Therapy, Research Center of Genome Medicine, Saitama Medical University, 1397-1 Yamane, Hidaka-shi, Saitama 350-1241, Japan; 4 Department of Dermatology, Keio University School of Medicine, 35 Shinanomachi, Shinjuku-ku 160-8582 Tokyo, Japan; 5 Department of Physiology, Keio University School of Medicine, 35 Shinanomachi, Shinjuku-ku 160-8582, Tokyo, Japan

**Keywords:** iPS cells, Retina, Neurodegeneration, Gene delivery, Drug screening, ER stress

## Abstract

**Background:**

Retinitis pigmentosa (RP) is an inherited human retinal disorder that causes progressive photoreceptor cell loss, leading to severe vision impairment or blindness. However, no effective therapy has been established to date. Although genetic mutations have been identified, the available clinical data are not always sufficient to elucidate the roles of these mutations in disease pathogenesis, a situation that is partially due to differences in genetic backgrounds.

**Results:**

We generated induced pluripotent stem cells (iPSCs) from an RP patient carrying a *rhodopsin* mutation (E181K). Using helper-dependent adenoviral vector (HDAdV) gene transfer, the mutation was corrected in the patient’s iPSCs and also introduced into control iPSCs. The cells were then subjected to retinal differentiation; the resulting rod photoreceptor cells were labeled with an *Nrl* promoter-driven enhanced green fluorescent protein (EGFP)-carrying adenovirus and purified using flow cytometry after 5 weeks of culture. Using this approach, we found a reduced survival rate in the photoreceptor cells with the E181K mutation, which was correlated with the increased expression of endoplasmic reticulum (ER) stress and apoptotic markers. The screening of therapeutic reagents showed that rapamycin, PP242, AICAR, NQDI-1, and salubrinal promoted the survival of the patient’s iPSC-derived photoreceptor cells, with a concomitant reduction in markers of ER stress and apoptosis. Additionally, autophagy markers were found to be correlated with ER stress, suggesting that autophagy was reduced by suppressing ER stress-induced apoptotic changes.

**Conclusion:**

The use of RP patient-derived iPSCs combined with genome editing provided a versatile cellular system with which to define the roles of genetic mutations in isogenic iPSCs with or without mutation and also provided a system that can be used to explore candidate therapeutic approaches.

## Background

Recent advances in molecular genetics have enabled the early diagnosis of neurodegenerative diseases, including retinitis pigmentosa (RP), an inherited retinal disorder that causes progressive photoreceptor cell loss and visual function impairment that can limit social activity and the ability to work. Despite its early detection, there is currently no cure for this disease.

Approximately 3,000 mutations have been reported in 50 genes in RP patients [1], and more than 100 point mutations have been identified in the *rhodopsin* gene [2]. Rhodopsin, an evolutionarily conserved seven-transmembrane protein specifically produced in photoreceptor cells, is first localized to the endoplasmic reticulum (ER) and is then transported to the outer segment discs where it responds to photon activation via conformational changes. Pathological responses to genetic mutations in *rhodopsin* typically occur in an autosomal dominant manner due to the production of an abnormal protein. Some types of abnormal rhodopsin proteins can be misfolded and retained in ER; in some cases, the mutant proteins are bound by the ER-resident chaperone, BiP [3]. The accumulated mutant proteins may induce unfolded-protein response (UPR) to alleviate the ER stress. In general, the abnormal proteins could be degraded through ubiquitin proteasome pathway and/or autophagy [4]. However, if the mutant protein was overloaded, the prolonged UPR will induce ER stress-associated programmed cell death, apoptosis [5]. Although many *rhodopsin* gene abnormalities are believed to be related to ER stress [3], practical therapies targeting mutant rhodopsin proteins or downstream signaling pathways have yet to be established. This may be due, in part, to the insufficient understanding of the disease pathogenesis: mutations associated with RP are genetically heterogeneous, and, in most cases, there is no formal proof of a causal relationship between the genetic mutation and the RP phenotype. Furthermore, only a limited number of genetic abnormalities have been reproduced and studied in *Drosophila*[6] and mouse systems [7,8], and drug screening is not easily performed due to the lack of appropriate screening systems. Although the abnormal gene of interest can be expressed in cell lines, overexpression commonly results in artificial cellular responses.

In an effort to develop an authentic cell-based model of human RP, induced pluripotent stem cell (iPSC) technology [9,10] has been recently applied to this disorder [11,12]. However, a causal relationship between genetic mutations and the RP phenotype remains to be elucidated. In the present study, we generated iPSCs from the somatic cells of an RP patient carrying a heterozygous mutation in the *rhodopsin* gene [13]. These cells were then differentiated into rod photoreceptor cells to investigate the cellular pathogenesis of RP and to screen chemical therapeutics. A comparison of the RP and control iPSC-derived photoreceptor cells showed that the RP patient’s iPSC-derived rod photoreceptor cells had a reduced survival rate in culture and an increased ER stress response. Furthermore, to formally demonstrate that the phenotype was due to the expression of mutant rhodopsin, we utilized the helper-dependent adenoviral vector (HDAdV) to replace the mutated *rhodopsin* gene in the RP patient’s iPSCs with the wild-type *rhodopsin* gene, thus repairing the gene, and found that the phenotype of the iPSC-derived photoreceptor cells reverted to normal. This method allowed a phenotypic comparison between the iPSC-derived photoreceptor cells of the same genetic background and developmental course during iPSC generation. Moreover, replacing the wild-type gene in the control iPSCs with a mutated gene using HDAdV reconstructed the pathological condition. We next used the RP patient’s iPSC-derived photoreceptor cells to screen for chemical reagents that rescued the ER stress phenotype. The involvement of autophagy, which can be induced in response to ER stress [14], was also explored.

## Results

### Generation of iPSCs from an RP patient

**Figure 1:**
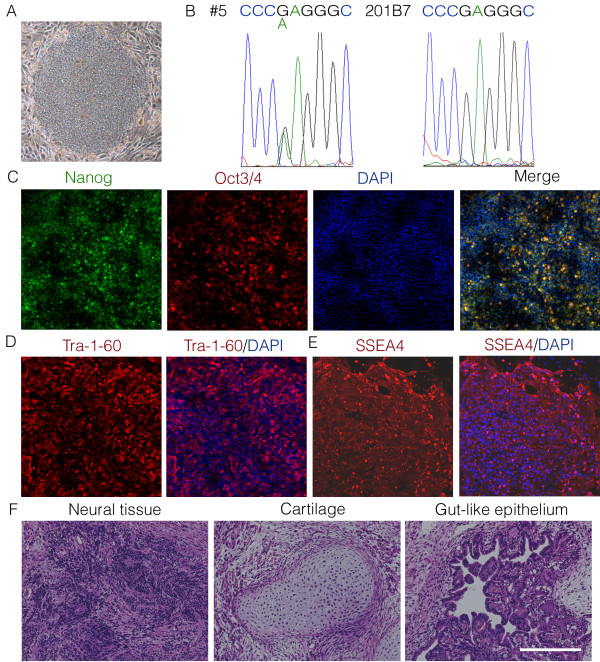
**RP patient’s iPSCs. (A)** A colony of RP#5 iPSCs derived from an RP patient’s skin cells. **(B)** DNA analysis of the *rhodopsin* gene in the RP#5 iPSCs and control 201B7 iPSCs. **(C-E)** Immunostaining for pluripotent markers; the nuclei were counterstained with DAPI (blue). **(C)** Nanog and Oct3/4. **(D)** Tra-1-60. **(E)** SSEA4. **(F)** Teratoma formation assay showing that the RP#5 iPSCs gave rise to all three germ layers, confirming their pluripotency. Scale bar, 1 mm **(A)**, 250 μm **(C-F)**.

The iPSC line RP#5 (#5) was generated using skin cells [15] isolated from an RP patient carrying a *rhodopsin* mutation (a G to A substitution at nucleotide 541) (Figure 1A) [13]. The point mutation resulted in a change in amino acid 181 from a glutamic acid (E) to lysine (K) (E181K) and was shown to be present on one allele in the #5 iPSCs but not in the 201B7 (B7) iPSCs (Figure 1B). The expression of pluripotent markers (Figure 1C-E) and the formation of teratomas containing all three germ layer cells (Figure 1F) were also confirmed.

### Preparation of gene-targeted iPSC lines

**Figure 2:**
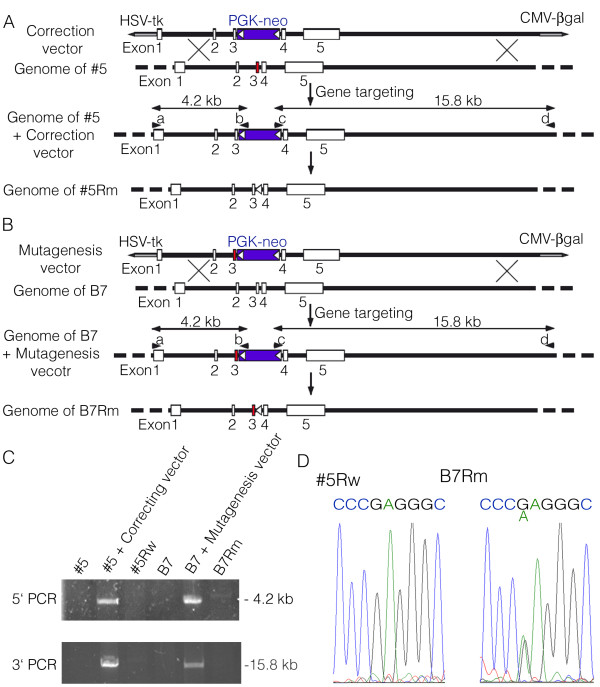
**The *rhodopsin *gene-targeting methods. (A, B)** Schematic illustrations of the *rhodopsin* gene correction implemented in the RP#5 iPSCs and the mutagenesis in the 201B7 iPSCs using HDAdVs. **(C)** A PCR analysis confirmed that recombination occurred at the *rhodopsin* locus. Products of 4.2 kb and 15.8 kb were obtained using primers a-b and c-d (arrowheads in **A**, **B**), respectively. **(D)** Sequence analysis of the recombinant *rhodopsin* genes in each iPSC line. HDAdV, helper-dependent adenoviral vector; HSV-tk, herpes simplex virus thymidine kinase gene cassette; *Neo*, neomycin-resistance gene cassette; white triangles, loxP sites; red boxes, exon 3 of *rhodopsin* containing the E181K mutation. #5, RP#5 iPSCs; #5Rw, *rhodopsin* gene-corrected RP#5 iPSCs; B7, 201B7 iPSCs; B7Rm, *rhodopsin* gene-mutated 201B7 iPSCs.

To determine whether the expression of rhodopsin E181K was solely responsible for the accelerated photoreceptor cell loss, we prepared *rhodopsin* gene-targeted iPSCs using HDAdVs. A wild-type *rhodopsin* gene in a BAC clone, with a *Neo* cassette introduced in the third intron, was inserted into an HDAdV vector to generate the correction vector (Figure 2A). Using this correction vector, the wild-type *rhodopsin* gene was replaced with the genome sequence of the #5 iPSCs through homologous recombination, followed by the removal of the *Neo* cassette by Cre recombinase to generate #5Rw iPSCs (Figure 2A). Similarly, the mutated *rhodopsin* sequence obtained from the genome of the #5 iPSCs was inserted into an HDAdV vector to construct a mutagenesis vector (Figure 2B) that was transferred into the genome of B7 iPSCs, followed by the removal of the *Neo* cassette, to generate B7Rm iPSCs (Figure 2B).

The introduction and removal of the *Neo* cassette at the *rhodopsin* locus were confirmed by PCR analyses (Figure 2C). We further confirmed the absence of the *rhodopsin* point mutation in the #5Rw cells and the presence of the heterozygous point mutation in the B7Rm cells (Figure 2D). These data indicated that the targeted *rhodopsin* gene correction and mutagenesis were successful.

### Impact of the *rhodopsin* gene mutation in differentiated rod photoreceptor cells derived from iPSC lines

**Figure 3:**
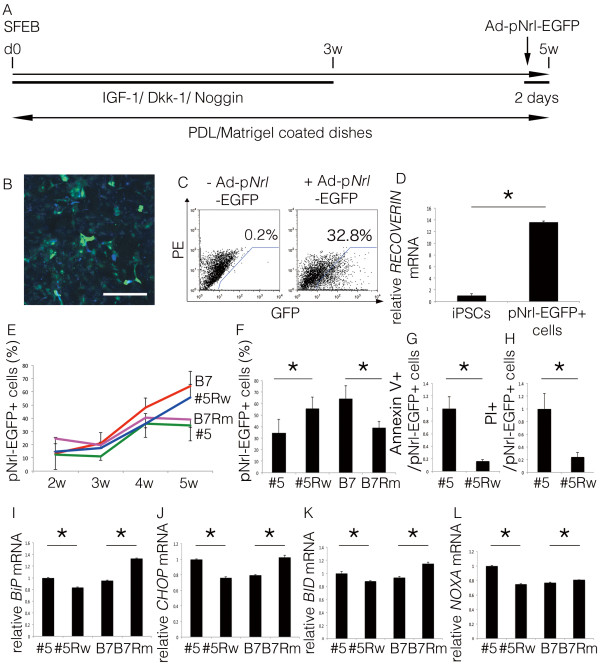
**Impact of the E181K *rhodopsin *gene mutation on rod photoreceptor cells derived from iPSC lines. (A)** Protocol of rod photoreceptor cell differentiation. **(B)** Expression of the rod photoreceptor cell-specific gene *Nrl* was visualized by infection with the Ad-p*Nrl*-EGFP virus. **(C)** Flow cytometry analysis of the differentiated cells without (left) and with (right) Ad-p*Nrl*-EGFP infection at 5 weeks. **(D)** The *recoverin* mRNA levels of the flow cytometry-purified p*Nrl*-EGFP-positive rod photoreceptor cells derived from the #5 iPSCs compared to the undifferentiated #5 iPSCs at the same time point. **(E)** Quantification of the p*Nrl*-EGFP-positive rod photoreceptor cells in each iPSC line after 2, 3, 4, and 5 weeks of differentiation. Red, B7; blue, #5Rw; pink, B7Rm; green, #5. N = 9. **(F)** Proportion of p*Nrl*-EGFP-positive rod photoreceptor cells derived from iPSC lines after 5 weeks. N = 9. **(G, H)** The ratio of dead cells in p*Nrl*-EGFP-positive photoreceptor cells detected by Annexin V **(G)** and PI **(H)**. N = 4. (I-L) Relative mRNA levels of BiP **(I)**, CHOP **(J)**, BID **(K)**, and NOXA **(L)** normalized to b-Actin in the p*Nrl*-EGFP-positive cells collected after 5 weeks, as determined by a real-time PCR analysis. N = 3. Ad-p*Nrl*-EGFP, adenovirus promoter *Nrl*-EGFP. *p < 0.05. Scale bar, 40 μm. Mean ± SD (with each p-values of marked by *) for iPSCs and p*Nrl*-EGFP cells in **(D)** 1 ± 0.38, 13.6 ± 0.14 (p = 0.049); for #5, #5Rw, B7, B7#Rm in **(F)** 34.6 ± 11.7, 55.7 ± 10.0 (p = 0.035), 64.3 ± 11.3, 38.9 ± 5.8 (p = 0.013); for #5 and #5Rw in **(G)** 1 ± 0.18, 0.16 ± 0.02, (p = 0.019) **(H)** 1 ± 0.24, 0.24 ± 0.07 (p = 0.021); for #5, #5Rw, B7, B7#Rm in **(I)** 1 ± 0.005, 0.83 ± 0.002 (p < 0.0001), 0.95 ± 0.006, 1.33 ± 0.004 (p < 0.0001); **(J)** 1 ± 0.002, 0.76 ± 0.007 (p < 0.0001), 0.79 ± 0.005, 1.03 ± 0.010 (p < 0.0001); **(K)** 1 ± 0.016, 0.88 ± 0.006 (p = 0.003), 0.94 ± 0.008, 1.14 ± 0.013 (p < 0.0001); **(L)** 1. ± 0.005, 0.75 ± 0.005 (p < 0.0001), 0.77 ± 0.005, 0.80 ± 0.004 (p = 0.001). All statistical analyses in this figure were carried out by Student’s T test.

Next, we induced retinal cell differentiation using the serum-free embryoid body (SFEB) method, along with subsequent stepwise changes in the culture medium for several weeks, as previously reported and modified by Lamba et al. [16]. iPSCs were cultured in the presence of Noggin, Dkk-1, and IGF-1 for 3 weeks, followed by 2 weeks of culture in their absence [16]. Using this method, up to 10% of the differentiated cells were reported to express early markers of photoreceptor differentiation at the end of 3 weeks, and these cells can be transplanted into subretinal space where they will integrate into the retina to form synapses [16-18]. In the present study, a recombinant adenovirus expressing EGFP under the control of the *neural retina leucine zipper* promoter, a rod photoreceptor-specific marker that acts as a transcription factor for the *rhodopsin* gene, (Ad-p*Nrl*-EGFP) [19-21], was introduced 2 days before flow cytometry analyses (Figure 3A, B). The specific detection of EGFP by flow cytometry was confirmed using the cells with (Figure 3C right) and without Ad-p*Nrl*-EGFP infection (Figure 3C left). This experiment also confirmed that 32.8% of the cells were Ad-p*Nrl*-EGFP positive after 5 weeks of culture. Among the Ad-p*Nrl*-EGFP-positive cells derived from the #5 iPSCs, we confirmed that recoverin, a photoreceptor marker, was upregulated after 5 weeks in differentiation culture compared to the non-differentiated #5 iPSCs (Figure 3D).

Using this method, rod photoreceptor cells derived from each iPSC line (#5, #5Rw, B7, and B7Rm) were quantified weekly from 2 to 5 weeks after differentiation was initiated (Figure 3E). During the first 4 weeks of culture, the proportion of Ad-p*Nrl*-EGFP-positive rod photoreceptor cells gradually increased with time, and there was no difference among the cell lines. However, after 5 weeks in differentiation culture, the proportion of rod photoreceptor cells was significantly higher in the cultures derived from the #5Rw and B7 iPSC lines, which did not contain the mutated *rhodopsin* coding sequences, compared to the #5 and B7Rm lines, which did contain the mutation (Figure 3E, F). Furthermore, we investigated the ratio of apoptotic cells of p*Nrl*-EGFP-positive rod photoreceptor cells derived from #5 and #5Rw iPSCs, by immunostaining using an anti-Annexin V antibody (Figure 3G) and by PI staining (Figure 3H). The p*Nrl*-EGFP-positive cells derived from #5 iPSCs included more apoptotic cells than those derived from #5Rw iPSCs, suggesting that the lower number of the rod differentiated photoreceptor cells derived from #5 than from #5Rw iPSCs was caused, at least in part, by the enhanced apoptosis of rod photoreceptor cells derived from #5 iPSCs. These data collectively indicated that the *rhodopsin* E181K mutation was solely responsible for the rod photoreceptor cell loss associated with this patient.

Because ER stress has been implicated in the pathogenesis of RP that involves *rhodopsin* mutations [3], we analyzed the expression of the ER stress markers BiP (Figure 3I) and CHOP (Figure 3J) using real-time PCR analyses. For this purpose, we purified the Ad-p*Nrl*-EGFP cells using flow cytometry and extracted mRNA from the Ad-p*Nrl*-EGFP-positive rod photoreceptor cells. The mRNA levels of both BiP and CHOP were elevated in the #5 and B7Rm iPSC-derived rod photoreceptor cells after 5 weeks in differentiation culture. Furthermore, we also examined the apoptosis-related molecules BID (Figure 3K) and NOXA (Figure 3L). After 5 weeks of culture, these molecules were also upregulated in the purified Ad-p*Nrl*-EGFP-positive rod photoreceptor cells derived from the #5 and B7Rm iPSCs, suggesting that the mutant rhodopsin protein induced ER stress and apoptosis.

### Drug screening using rod photoreceptor cells derived from RP iPSCs

**Figure 4:**
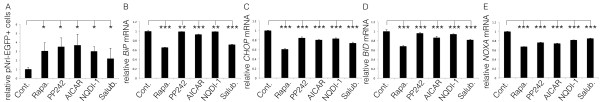
**Drug screening in the RP #5 iPSC-derived rod photoreceptor cells. (A)** Relative number of p*Nrl*-EGFP-positive rod photoreceptor cells derived from #5 iPSCs after treatment with each therapeutic reagent. N = 9. **(B-E)** Relative mRNA levels of BiP **(B)**, CHOP **(C)**, BID **(D)**, and NOXA **(E)** in the p*Nrl*-EGFP-positive cells cultured with rapamycin, PP242, AICAR, NQDI-1, and salubrinal at 5 weeks after differentiation. Each reagent increased the rod photoreceptor cell survival at 5 weeks, whereas ER stress and apoptotic markers were suppressed. N = 3 for **B**-**E**. Rapa., rapamycin; Salub., salubrinal. *p < 0.05, **p < 0.01, ***p < 0.001. Mean ± SD relative to cont. (with each p-values of marked by *) for Cont., Rapa., PP242, AICAR, NQDI-1, Salbr. in **(A)** 1 ± 0.182, 3.02 ± 0.920, 3.49 ± 0.976, 3.67 ± 1.22, 2.99 ± 0.513, 2.19 ± 1.12 (all, p < 0.0001); **(B)** 1 ± 0.005, 0.654 ± 0.001, 0.990 ± 0.001, 0.932 ± 0.001, 0.989 ± 0.001, 0.714 ± 0.004 (all, p < 0.0001); **(C)** 1 ± 0.002, 0.608 ± 0.003, 0.842 ± 0.009, 0.802 ± 0.003, 0.830 ± 0.003, 0.733 ± 0.008 (all, p < 0.0001); **(D)** 1 ± 0.016, 0.681 ± 0.006, 0.954 ± 0.007, 0.864 ± 0.002, 0.934 ± 0.004, 0.816 ± 0.008 (all, p < 0.0001); **(E)** 1 ± 0.005, 0.671 ± 0.001, 0.760 ± 0.004, 0.743 ± 0.003, 0.816 ± 0.001, 0.849 ± 0.006 (all, p < 0.0001). These data were obtained by technical triplicate and not by biological triplicate. All statistical analyses in this figure were carried out by One-way ANOVA Dunnett’s test.

To explore treatments that may protect rod photoreceptor cells from the accelerated cell loss induced by the *rhodopsin* mutation, we treated the cells with reagents that could modify ER stress-related pathways and quantified the Ad-p*Nrl*-EGFP-positive rod photoreceptor cells using flow cytometry after 5 weeks in differentiation culture. The reagents were added to the medium after 3 weeks of culture and were re-added each time the medium was changed (every 2–3 days). After 5 weeks of culture, the number of #5 iPSC-derived rod photoreceptor cells collected and counted by flow cytometry was significantly increased following treatment with rapamycin and PP242 (both mTOR inhibitors), AICAR (an activator of AMP kinase [AMPK]), NQDI-1 (an inhibitor of apoptosis signal-regulating kinase 1 [ASK1]), and salubrinal (an inhibitor of eukaryotic translation initiation factor 2 subunit α [eIF2α] phosphatase and protein synthesis) (Figure 4A). These data showed that the E181K mutant rhodopsin-related cell loss could be suppressed by mTOR inhibition, AMPK activation, ASK1 inhibition, or the suppression of protein synthesis.

### Effect of treatments on ER stress and apoptosis markers

Next, we investigated the effects of reagents on ER stress markers in the #5 iPSC-derived rod photoreceptor cells. After the Ad-p*Nrl*-EGFP-positive cells were purified and treated with the above-mentioned reagents, mRNA was harvested from the cells and analyzed using real-time PCR. The mRNA levels of BiP and CHOP were found to be reduced following rapamycin, PP242, AICAR, NQDI-1, or salubrinal treatment (Figure 4B, C) in the Ad-p*Nrl*-EGFP-positive rod photoreceptor cells, suggesting that these reagents suppressed the ER stress caused by the mutant rhodopsin. Additionally, the expression levels of apoptosis-related molecules, which were upregulated in the rod photoreceptor cells expressing the mutant rhodopsin, were decreased following the addition of these same drugs (Figure 4D, E).

### Involvement of autophagy

**Figure 5:**
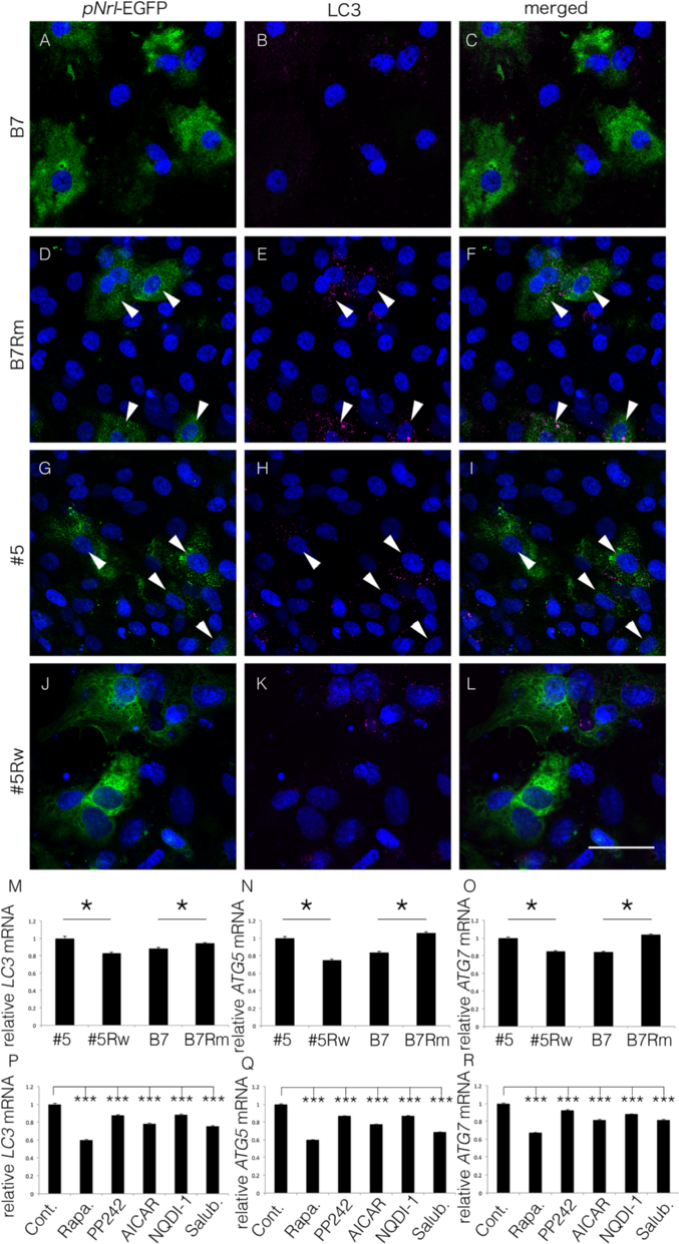
**Autophagy markers in iPSC-derived rod photoreceptor cells.** Immunostaining of p*Nrl*-EGFP-positive cells (green: **A**, **D**, **G** and **J**) and immunocytochemistry using anti-LC3 antibody (magenta: **B**, **E**, **H** and **K**), and their merged images **(C, F, I, L)**. Arrowheads indicated p*Nrl*-EGFP- and LC3-double positive cells. Scale bar: 20 μm. The autophagy marker molecules LC3 **(M, P)**, ATG5 **(N, Q)**, and ATG7 **(O, R)** were analyzed using real-time PCR in the p*Nrl*-EGFP-positive rod photoreceptor cells derived from each iPSC line in the absence of treatment **(M-O)** and in #5 iPSC-derived cells following therapeutic treatment **(P-R)**, both at 5 weeks of culture. Autophagy was suppressed in the #5Rw and B7 iPSC-derived cells and in the #5 iPSC-derived cells with each treatment. N = 3 for all. Rapa., rapamycin; Salub., salubrinal. *p < 0.05, ***p < 0.001. Mean ± SD (with each p-values of marked by *) for #5, #5Rw, B7, B7#Rm in **(M)** 1 ± 0.010, 0.830 ± 0.005 (p < 0.0001), 0.881 ± 0.008, 0.943 ± 0.003 (p = 0.002); **(N)** 1 ± 0.007, 0.747 ± 0.005 (p < 0.0001), 0.836 ± 0.005, 1.06 ± 0.006 (p < 0.0001); **(O)** 1 ± 0.004, 0.849 ± 0.003 (p < 0.0001), 0.842 ± 0.003, 1.04 ± 0.003 (p < 0.0001). Mean ± SD relative to cont. (with each p-values of marked by *), for Cont.. Rapa., PP242, AICAR, NQDI-1, Salbr. for **(P)** 1 ± 0.010, 0.601 ± 0.001, 0.873 ± 0.011, 0.784 ± 0.004, 0.885 ± 0.004, 0.757 ± 0.002 (all, p < 0.0001); **(Q)** 1 ± 0.007, 0.599 ± 0.001, 0.867 ± 0.004, 0.772 ± 0.003, 0.872 ± 0.0003, 0.683 ± 0.001 (all, p < 0.0001); **(R)** 1 ± 0.004, 0.675 ± 0.001, 0.924 ± 0.003, 0.818 ± 0.002, 0.879 ± 0.001, 0.818 ± 0.004 (all, p < 0.0001). These data were obtained by technical triplicate and not by biological triplicate. Statistical analyses in **M**-**O** and **P**-**R** were carried out by Student’s T test and by One-way ANOVA Dunnett’s test, respectively.

As ER stress is known to activate autophagy to overcome cellular dysfunction, we examined autophagy markers in each line of iPSC-derived rod photoreceptor cells in the presence and absence of treatment with different drugs. We first examined LC3 immunostaining that indicates a putative autophagosome in p*Nrl-*EGFP-positive photoreceptor cells (Figure 5A-L), and found that the presence of LC3 was obvious in the cells with *rhodopsin* mutation (Figure 5D-I), but hardly detected in the cells without the mutation (Figure 5A-C, J-L). The autophagy markers LC3, Atg5, and Atg7 were all suppressed in the #5Rw and B7 iPSC-derived rod photoreceptor cells compared to the levels in the #5 and B7Rm iPSC-derived cells (Figure 5M-O). Treatment of the #5 iPSC-derived rod photoreceptor cells with each of the reagents described above also resulted in the reduced expression of the autophagy markers LC3, ATG5, and ATG7 (Figure 5P-R).

## Discussion

We generated an iPSC line from the somatic cells of a patient with RP who carried the *rhodopsin* E181K mutation, and this iPSC line was used to derive rod photoreceptor cells that harbored the same *rhodopsin* mutation. These cells were then used to demonstrate that the E181K mutation was indeed a pathogenic, disease-causing mutation and were used to explore the underlying molecular mechanisms and potential therapeutic approaches.

A considerable number of genetic abnormalities are recognized as the cause of RP pathogenesis. Although multiple genes and multiple mutations within these genes have been linked to RP, some of these mutations may, in fact, be non-pathogenic, and, in some cases, patients may have more than one mutation in their genome [1]. Moreover, RP is exceptionally heterogeneous, and the same mutation in different individuals may produce different clinical consequences due, in part, to the different genetic backgrounds of the individuals [1]. Given these complications, it has been challenging to determine the precise genotype-phenotype association of a large number of mutations. In the present study, using human iPSCs and gene manipulation, we demonstrated that the correction of a *rhodopsin* gene mutation reversed photoreceptor cell loss in the iPSC-derived rod photoreceptor cells of an RP patient, whereas mutagenesis of the *rhodopsin* gene in control iPSCs increased cell loss. These experiments directly demonstrated the pathogenicity of the *rhodopsin* mutation in an *in vitro* system. When utilizing iPSCs to analyze disease pathogenesis, there may be a concern that the observed phenotype might be related to differences in the cell lines that reflect differences in the reprogramming process [22]. Thus, to exclude such a concern, we investigated the effect of correcting the genetic defect in the patient-derived iPSC line, aiming to clarify the genotype-phenotype causal relationship.

This genetically well-controlled study was facilitated by the use of HDAdV for the gene targeting of human iPSCs. HDAdV was originally developed to overcome host immune responses to E1-deleted AdV, the adenovirus commonly used for gene transfer [23]. Because the viral genes are completely removed from the vector genome, the HDAdV system is less toxic to the infected cells. Moreover, the increased cloning capacity of HDAdV when combined with negative selection was shown to result in an increased frequency of targeted integrations in human iPSCs [24]. In the present study, the use of this methodology resulted in the successful generation of targeted iPSCs, and large gene targeting in iPSCs will be useful for establishing the pathogenesis of various candidate genes associated with hereditary diseases [25]. Moreover, because HDAdV gene transfer does not result in the transfer of viral sequences, this technique may also have the potential to be used for gene therapy via iPSC transplantation. Indeed, the HDAdV gene transfer system has several advantages compared to other genome-editing methods, such as CRISPR (clustered regularly interspaced short palindromic repeats) or TALEN (Transcription Activator-Like Effector Nucleases), which may induce off-target alterations [26]. Fu et al. reported that the off-target sites caused by CRISPR harbored up to five mismatches, and many sites were mutagenized with frequencies comparable to those observed at the intended on-target site. This is because the DNA break caused by Cas9 nuclease, which leads to the genome editing, can be guided by simple base-pair complementarity between the first 20 nucleotides of an engineered guide RNA-target DNA interface and can be easily misguided by sensing mismatched sequences. Undesired off-target sites when using TALEN are also related to unintended DNA cleavage [27]. In contrast, the HDAdV gene transfer system does not require DNA cleavage but instead requires homologous recombination; therefore, this technique results in few off-target effects.

Clinical trials using several therapeutic approaches for RP are currently in progress. One example is retinal pigment epithelium-specific 65-kDa protein (*RPE65*) gene therapy for the treatment of Leber’s congenital amaurosis (LCA); this autosomal recessive abnormality is caused by a loss-of-function of RPE65 and can thus be treated through the introduction of the normal gene.

In contrast, a different approach is required for autosomal dominant diseases caused by mutations that result in a toxic gain-of-function protein. A randomized trial of ciliary neurotrophic factor (CNTF) was performed to evaluate the safety and efficacy of this factor with regard to the visual functions of RP patients [28]. Although this treatment caused no serious adverse events, retinal sensitivity was reduced, possibly due to rhodopsin degradation [29] in response to CNTF. Thus, definitive therapeutic approaches for RP have not yet been established. Our study using a patient’s iPSC-derived photoreceptor cells offers a novel approach for the evaluation of potential of new therapeutics.

We found that treatment with salubrinal, a selective inhibitor of eIF2α, led to an increased number of #5 iPSC-derived rod photoreceptor cells. Additionally, the treated cells showed reduced levels of ER stress and apoptotic markers, suggesting that the rod photoreceptor cell death caused by the *rhodopsin* E181K mutation could be suppressed by inhibiting protein synthesis, including the synthesis of the abnormal rhodopsin. Treatment with NQDI-1, an inhibitor of ASK1 activation, also increased the survival of the mutant rod photoreceptor cells, consistent with the idea that apoptosis is regulated by the ER stress-induced Ire-1α-ASK1-JNK pathway [30,31].

Based on these findings, we further investigated the ER stress-induced apoptosis pathway using additional reagents that modify this signaling pathway. Treatment with rapamycin reduced ER stress markers in the #5 iPSC-derived rod photoreceptor cells and significantly increased cell survival. The accumulation of unfolded mutant rhodopsin protein, which increases ER stress, may have activated the mTORC1-regulated Ire1α-ASK1-JNK apoptotic pathway [30]; mTORC1 can further increase cell death through a positive feedback mechanism, resulting in increased protein synthesis, including the mutated rhodopsin [30]. Thus, the protective effect of rapamycin in these cells may be due to the suppression of the vicious cycle between the UPR (unfolded protein response) and mTORC1 pathways. This FDA-approved drug (rapamycin) can be reassessed to treat RP; however, further studies are required. In contrast to rapamycin, PP242 inhibits both mTORC1 and mTORC2; the latter is also influenced by UPR, and mTORC2 signaling induces survival signaling via AKT activation [30]. This contradictory action of mTORC2 may limit the overall effect of PP242 on the suppression of cell death.

AMPK activation through AICAR treatment also exhibited a protective effect by reducing ER stress and increasing photoreceptor cell survival. Previous studies have shown that AMPK suppresses mTORC1 indirectly through the phosphorylation and activation of the tuberous sclerosis complex (TSC) [30,32]. Additionally, extensive studies have also revealed that the activity of mTORC1 is modulated by intracellular energy levels through multiple mechanisms, and AMPK is reported to directly phosphorylate multiple components of the mTORC1 pathway [32].

Autophagy is a process that involves the degradation of proteins and organelles in response to various forms of cellular stress, including ER stress [14]. The unfolded or misfolded proteins in the ER lumen that cause ER stress are translocated to the cytoplasm where they are degraded. During this process, the ubiquitin proteasome system and autophagy act as degradation systems for the unfolded proteins. Thus, disturbing autophagy renders the cells vulnerable to ER stress, as autophagy plays important roles in cell survival after ER stress [15]. The absence of autophagy may cause neurodegenerative diseases [33,34], and autophagy has also been shown to cause apoptosis in some diseases by destroying cellular components [35]. Accordingly, we examined the expression of autophagy markers in the iPSC-derived rod photoreceptor cells with or without the *rhodopsin* mutation. We found that the levels of autophagy markers changed in parallel with the levels of ER stress and the levels of the apoptosis markers. We interpret this result that the decrease in autophagy markers following the drug treatments may have resulted from the decreased demand for autophagy following the suppression of ER stress.

In the present study, to examine the effects of various drugs, and characterize the ER stress and autophagy marker expression, we could obtain consistent results with low p-values in the triplicated real-time PCR experiment (Figures 4 and 5). Strictly, however, these results can be interpreted as follows. Because of the limitation of the culture scale, we had to put each iPSC-derived p*NrL*-GFP positive rod photoreceptor cells which were obtained from each culture well together, before reverse transcription. Thus, each real-time PCR data in Figures 4 and 5 was obtained using the same RT-product as a template, rather than biological triplicate. These experimental conditions can explain why such low p-values for the mRNA expression changes by various drugs’ treatment. Thus, in the future investigations, these results should be re-confirmed using biological triplicate by performing larger scale of iPSC cultures and subsequent photoreceptor differentiation assays.

To obtain target cells that could be used to explore different therapeutic approaches, we used Lamba’s differentiation method [16], which allows the cells to express rod photoreceptor cell markers within only a few weeks. Considering that the human photoreceptor require more time to mature *in vivo*, it is possible that the photoreceptor cells derived using this method have artificial intracellular microenvironments. In fact, there are several protocols for retinal differentiation that involve months of culture [11,12,36,37]. However, the method used in the present study required less time and constitutes an efficient strategy for creating cells that can be used to examine pathogenic genes and screen novel therapeutics, which can be then applied in industrial uses.

## Conclusions

In summary, the generation of iPSCs from an RP patient was a valuable approach to demonstrate a causative link between a pathogenic mutation and a cellular phenotype. The use of iPSCs derived from RP and control individuals, combined with the manipulations of the gene of interest using HDAdV, allowed us to examine the effects of normal and mutant rhodopsin in otherwise genetically identical rod photoreceptor cells. Further studies using similar systems should help to reveal the molecular mechanisms underlying other genetic diseases and could serve as a cellular platform for the evaluation of potential therapeutics, including the large-scale screening of compound libraries.

## Methods

This study followed the tenets of the Declaration of Helsinki and was approved by the ethics committee at Keio University School of Medicine (Approval No. 2008016).

### Isolation of human skin cells and generation of iPSCs

Skin cells were obtained from a 53-year-old Japanese female RP patient by a skin-punch biopsy after the patient gave her written, informed consent. These cells were then infected with retroviruses encoding four reprogramming factors, Oct3/4, Sox2, Klf4, and c-Myc, as previously described, to generate a human iPSC line, RP#5 (#5), [15,38]. The control iPSC line [201B7 (B7)], which was generated using the same method described for #5, was kindly provided by Dr. Shinya Yamanaka of Kyoto University [15]. The sequences of the *rhodopsin* genes in the iPSCs (see Additional file 1: Table S1) and the immunostaining of pluripotent markers (see Additional file 2: Table S2) were analyzed; teratoma formation was confirmed as previously described [38].

### Preparation of HDAdVs and gene targeting

HDAdVs were prepared as previously described to generate gene-targeted iPSCs (#5Rw and B7Rm clones) (see Results and Additional file 3).

### Differentiation, collection, and analyses of rod photoreceptor cells

The *in vitro* differentiation of the rod photoreceptor cells from iPSCs was performed as previously reported (see Additional file 3) [16]. The differentiated cells were infected with Ad-p*Nrl*-EGFP, which was generated using the pENTR1A plasmid harboring the *Nrl* promoter region (kindly provided by Dr. Anand Swaroop, NIH, MD) [19], 2 days prior to each analysis. The cells were suspended in PBS containing 10 μg/ml propidium iodide (PI) to stain the non-viable cells and were sorted to collect the EGFP-positive viable cells using a triple-laser MoFlo (Dako), FACS Calibur or FACS Aria (BD Biosciences) flow cytometer. The collected cells were counted or used for a real-time PCR analysis (see Additional file 1: Table S1). For real-time PCR analyses, the differentiated cells were summed up from each culture well according to the iPSC groups or the treatment groups. Annexin V staining was performed using Annesin V-Biotin Apoptosis Detection Kit (Bio Vision), followed by the staining with Streptoavidin, Allophcocyanin, crosslinked, conjugated antibody (Life Technoloties). Immunohistochemical analyses were performed using antibodies listed in Additional file 2: Table S2. Images were obtained using LSM-710 confocal (Zeiss) microscopes.

### Treatment protocol

The iPSC-derived cells were treated with the following drugs after 3 weeks of differentiation: 10 nM rapamycin (Selleckchem.com), 500 nM PP242 (Sigma-Aldrich), 2 μM 5-aminoimidazole-4-carboxyamide ribonucleoside (AICAR, Santa Cruz), 500 nM Nuclear Quality Assurance-1 (NQDI-1, Axon Medchem), and 3 μM salubrinal (Millipore).

### Statistical analyses

All the results are expressed as the mean ± SD. The differences were analyzed using the Student’s T test (between 2 groups) and Dunnett’s test (among 6 groups), and the differences were considered significant when p < 0.05. All statistical tests were performed using IBM SPSS statistics Ver.19 (IBM, Armonk, NY) and confirmed using Stata13 (Light Stone, Tokyo, Japan).

## Competing interests

H. Okano is the scientific consultant of San Bio, Inc; Eisai Co Ltd; and Daiichi Sankyo Co Ltd.

## Authors’ contributions

TY, YO, SS, KT, and HO conceived and designed the experiments. TY performed most of the experiments, analyzed the data, and wrote the manuscript. YO and HO edited the manuscript. YO, WA, and MO generated the patient-derived iPSCs. KS and KM generated the genetically modified iPSCs. KY performed the statistical analyses and some of the *in vitro* culture assays. YM performed the flow cytometric analyses. All the authors read and approved the final manuscript.

## Supplementary Material

Additional file 1: Table S1 Primer list. Click here for file

Additional file 2: Table S2 Antibody list. Click here for file

Additional file 3 Supplementary materials and methods. Click here for file
