# Supplementary material for: The use of induced pluripotent stem cells to reveal pathogenic gene mutations and explore treatments for retinitis pigmentosa
Source: Mol Brain. 2014 Jun 16;7:45. doi: 10.1186/1756-6606-7-45 (PMC4058693; doi:10.1186/1756-6606-7-45)
Supplement: Additional file 1: Table S1 — Primer list. [file 1756-6606-7-45-S1.docx]

| Additional file 1: Table S1 Primer list. | |
| --- | --- |
|  |  |
| Primers for HDAdV preparation and sequencing | |
| *rhodopsin* 5’-primer-F | tgtacaaacttcctcaaatg |
| *rhodopsin* 5’-primer-R | ctggaccctcagagccgtga |
| *rhodopsin* 3’-primer-F | cccccagcatgcatctgcgg |
| *rhodopsin* 3’-primer-R | aagtagcttgtccttggcag |
| KI-primer-a | caagtcatgcagaagttagg |
| KI-primer-b | caagtcatgcagaagttagg |
| KI-primer-c | caagtcatgcagaagttagg |
| KI-primer-d | ccttctatcgccttcttgac |
| Neo-primer-F | tcggctatgactgggcacaacaga |
| Neo-primer-R | gggagcggcgataccgtaaagcac |
|  |  |
| Primers used for real-time PCR | |
| RECOVERIN-F | CCAGAGCATCTACGCCAAGT |
| RECOVERIN-R | CACGTAGTAGAGGGAGAAGG |
| BiP-F | CCTGGGTGGCGGAACCTTCGATGTG |
| BiP-R | CTGGACGGGCTTCATAGTAGACCGG |
| CHOP-F | GCCTTTCTCTTCGGACACTGTCAGC |
| CHOP-R | CTCGGCGAGTCGCCTCTACTTCCC |
| BID-F | GCTTCCAGTGTAGACGGAGC |
| BID-R | GTGCAGATTCATGTGTGGATG |
| Noxa-F | ACTGTTCGTGTTCAGCTC |
| Noxa-R | GTAGCACACTCGACTTCC |
| LC3-F | ATGCCGTCGGAGAAGACCTT |
| LC3-R | TTACACTGACAATTTCATCCCG |
| Atg5-F | TTGACGTTGGTAACTGACAAAGT |
| Atg5-R | TGTGATGTTCCAAGGAAGAGC |
| Atg7-F | GATCCGGGGATTTCTTTCACG |
| Atg7-R | CAGCAATGTAAGACCAGTCAAGT |
| b-Actin-F | GATCAAGATCATTGCTCCTCCT |
| b-Actin-R | GGGTGTAACGCAACTAAGTCA |
