# Supplementary material for: The use of induced pluripotent stem cells to reveal pathogenic gene mutations and explore treatments for retinitis pigmentosa
Source: Mol Brain. 2014 Jun 16;7:45. doi: 10.1186/1756-6606-7-45 (PMC4058693; doi:10.1186/1756-6606-7-45)
Supplement: Additional file 2: Table S2 — Antibody list. [file 1756-6606-7-45-S2.docx]

| Additional file 2: Table S2 Antibody list. | |
| --- | --- |
|  |  |
| Primary antibodies |  |
| Nanog | 1:1000, ReproCELL |
| Oct3/4 | 1:200, Santa Cruz Biotechnology |
| SSEA-4 | 1:200, Millipore |
| Tra-1-60 | 1:200, Millipore |
| LC3 | 1:1000, MBL |
| GFP | 1:1000, MBL |
|  |  |
| Secondary antibodies | |
| Alexa Fluor 488-conjugated antibody | 1:1000, Life Technologies |
| Alexa Fluor 555-conjugated antibody | 1:1000, Life Technologies |
|  |  |
| *Nuclei were visualized by DAPI staining. | |
