# Supplementary material for: The use of induced pluripotent stem cells to reveal pathogenic gene mutations and explore treatments for retinitis pigmentosa
Source: Mol Brain. 2014 Jun 16;7:45. doi: 10.1186/1756-6606-7-45 (PMC4058693; doi:10.1186/1756-6606-7-45)
Supplement: Additional file 3 — Supplementary materials and methods. [file 1756-6606-7-45-S3.docx]

**Additional file 3: Supplementary materials and methods.**

**Differentiation of rod photoreceptor cells**

The dissociated iPSCs were plated on Matrigel-coated dishes and cultured for 3 days in DMEM/F12 medium supplemented with N2 (Wako), B27 supplement (Miltenyi Biotech), glutamic acid (Wako), and NEAA (Sigma), together with 1 ng/ml mouse Noggin (R&D Systems), 1 ng/ml human recombinant Dkk-1 (R&D Systems), and 1 ng/ml human recombinant insulin-like growth factor-1 (IGF-1) (R&D Systems). Starting on day 4, the concentrations of the latter 3 factors were increased to 10 ng/ml each for 3 weeks; the factors were the removed from the medium. The medium was changed every 2-3 days.

**Construction and preparation of gene-targeting HDAdVs**

HDAdVs were constructed and prepared as previously described (1, 2). First, the 5’ homologous arms of the WT or mutant *rhodopsin* gene were amplified using 5’-primer-F and 5’-primer-R, and the 3’ homologous arm was amplified using 3’-primer-F and 3’-primer-R. The wild-type gene was amplified from a BAC clone (3), whereas the mutant gene was amplified from the #5 iPSC genome. A fragment of the PGK-*Neo*-pA cassette (Biological Resources Branch), which was sandwiched by loxP sites, was inserted into each amplified *rhodopsin* gene between exons 3 and 4. This fragment was then transferred to the BAC clone by homologous recombination in *E. coli*. Next, the 19.3-kb region including the *Neo* cassette was inserted into the HDAdV plasmid (1), and the constructs were propagated by serial passage in 293FLPe cells with the addition of the FL helper virus (kindly provided by Dr. Pedro Lowenstein, University of Manchester); the viruses were purified as previously described (4).

**Gene targeting**

iPSCs were infected with the constructed HDAdVs, and selection with 50 μg/ml G418 (Nacalai) and 2 μmol/l GANC (Invitrogen) was performed. The genomic DNA of the drug-resistant clones was screened by PCR using KI-primers a-d (Supplementary table 1). The cells were then dissociated into single cells in the presence of the Rho-associated coiled kinase (ROCK) inhibitor Y-27632 (10 μmol/l; Wako) and then infected with a recombinant adenovirus harboring a CAG-Cre construct (kindly provided by Dr. Yumi Kanegae of Tokyo University). The removal of the *Neo* cassette was confirmed by G418 sensitivity and PCR using the following primers: Neo-primer-F and Neo-primer-R (Supplementary table 1).

**Real-time RT-PCR**

The differentiated rod photoreceptor cells were collected by flow cytometry using p*Nrl*-EGFP as an indicator. Total RNA was isolated using an RNeasy Micro Kit (Qiagen). Complementary DNA (cDNA) was generated by adding 1 μg of total RNA to the High Capacity RNA-to-cDNA Master Mix (Applied Biosystems) and was reverse-transcribed according to the manufacturer’s instructions. The mRNA levels were normalized to the levels of β-actin. PCR was performed using the StepOnePlus Real Time PCR system (Applied Biosystems), and the delta CT method was used to quantify gene expression. The primers are shown in Supplementary table 1.

**References**

1. Aizawa E, Hirabayashi Y, Iwanaga Y, Suzuki K, Sakurai K, Shimoji M, Aiba K, Wada T, Tooi N, Kawase E, Suemori H, Nakatsuji N, Mitani K (2012) Efficient and accurate homologous recombination in hESCs and hiPSCs using helper-dependent adenoviral vectors. *Mol Ther* 20(2):424-431.
2. Liu GH, Suzuki K, Qu J, Sancho-Martinez I, Yi, F, Li M, Kumar S, Nivet E, Kim J, Soligalla d, Dubova I, Goebl A, Plongthongkum N, Fung HL, Zhang K, Loring JF, Laurent LC, Belmonte JC (2011) Targeted gene correction of laminopathy-associated LMNA mutations in patient-specific iPSCs. *Cell Stem Cell* 8(6):688–694.
3. Osoegawa K, Mammoser AG, Wu C, Frengen E, Zeng C, Catanese JJ, de Jong PJ (2001) A Bacterial Artificial Chromosome Library for Sequencing the Complete Human Genome. *Genome Res* 11(3):483-496.
4. Umaña P, Gerdes CA, Stone D, Davis JR, Ward D, Castro MG, Lowenstein PR (2001) Efficient FLPe recombinase enables scalable production of helper-dependent adenoviral vectors with negligible helper-virus contamination. *Nat Biotechnol* 19(6):582-585.
